# A Multicarbohydrase and Phytase Complex Is Able to Compensate a Nutrient-Deficiency in Growing-Finishing Pigs

**DOI:** 10.3390/ani11041129

**Published:** 2021-04-15

**Authors:** Ya-Kuan Huang, Ling Zhao, Hua Sun, Xue-Mei Xu, Jlali Maamer, Aurélie Preynat, Lv-Hui Sun, De-Sheng Qi

**Affiliations:** 1 Department of Animal Nutrition and Feed Science, College of Animal Science and Technology, Huazhong Agricultural University, Wuhan 430070, China; huangyk@newhope.cn (Y.-K.H.); zling@mail.hzau.edu.cn (L.Z.); huasun@webmail.hzau.edu.cn (H.S.); xuxm@vlandgroup.com (X.-M.X.); 2 Center of Expertise and Research in Nutrition, Adisseo France SAS, F-03600 Commentry, France; aurelie.preynat@adisseo.com

**Keywords:** multi-carbohydrase and phytase complex, fecal digestibility, ileal digestibility, nutrient, phytate, pig

## Abstract

**Simple Summary:**

Phytate is the primary storage form of phosphorus in grain-based feedstuffs, which can reduce the utilization of the phosphorus, calcium, and other minerals. Additionally, non-starch polysaccha-rides can increase digesta viscosity and thus decrease the nutrient digestion and utilization. The current study has evaluated the effects of a next-generation multicarbohydrase and phytase com-plex on the growth performance, apparent total tract digestibility of nutrients, carcass traits, and meat quality in growing-finishing pigs fed a corn-wheat-soybean meal-based diet. The results showed that dietary supplementation of the multicarbohydrase and phytase complex improved the growth performance and nutrient digestibility but had little effect on carcass traits and meat quality in growing-finishing pigs fed a corn-soybean meal-wheat-based diet. These findings indi-cate that the multicarbohydrase and phytase complex could be used as a promising enzymes product to mitigate the negative effects of phytate and non-starch polysaccharides.

**Abstract:**

The objective of this study was to evaluate the efficacy of supplementing a corn-wheat-soybean meal-based diet with a multicarbohydrase and phytase complex (MCPC) on growth performance, apparent total tract digestibility (ATTD) of nutrients, carcass traits, and meat quality in growing-finishing pigs. A total of 300 pigs (Duroc × Large White × Landrace; body weight = 25.3 ± 0.7 kg) were randomly allotted to three groups with 10 replicates of 10 pigs each. Pigs from three groups were fed positive control (PC) or negative control (NC), without or with MCPC diets, respectively. The MCPC supplied at least 1800, 1244, 6600, and 1000 units of xylanase, β-glucanase, α-arabinofuranosidase, and phytase per kilogram of diet, respectively. The NC diet was the PC diet but reduced in net energy (NE), digestible amino acids (dig. AA), digestible P (dig. P), and Ca by 74 kcal/kg, 7.0%, 0.134, and 0.119 percentage points, respectively. The diets were fed in 4 growth phases based on body weight (BW): phase 1: 25–50 kg, phase 2: 50–75 kg, phase 3: 75–100 kg, and phase 4: 100–135 kg. Compared to the PC, the NC diet decreased (*p* < 0.05) body weight gain, feed intake, and(or) feed to gain ratio during the growing/finishing phases 1, 2, 3, and 4. It also reduced (*p* < 0.05) the ATTD of crude protein, crude fat, P, and Ca of pigs. MCPC supplementation improved (*p* < 0.05) the body weight gain, feed intake, and(or) feed to gain ratio in phases 2, 3, and 4 and the ATTD of crude protein, crude fat, ash, P, and Ca for the NC diet. Additionally, dietary treatment had no effects on carcass traits and meat quality with the exception that the loin eye area in the NC plus MCPC diet was higher (*p* < 0.05) than the NC diet. In conclusion, the addition of MCPC to a corn-soybean meal-wheat-based diet reduced in energy and nutrients improved the growth performance and nutrient digestibility but had little effect on carcass traits and meat quality in growing-finishing pigs.

## 1. Introduction

In the grains with their byproducts and oil cakes, 65 to 75 percent of phosphorus (P) is in the form of phytate (myo-inositol hexaphosphate, IP_6_) [1]. Pigs have a poor utilization of P from IP_6_ due to lack of significant endogenous activity in the digestive tract to efficiently dissociate phosphate radical from organic compounds [2,3]. However, adding phytase to the diet with high IP_6_ content can greatly improve the utilization rate of P [4,5]. In addition, arabinoxylan chains are the main non-starch polysaccharides (NSP) in corn and wheat, reaching up to 4.7% and 7.3% dry matter, respectively [6]. The concentration of β-glucan ranges from 1.0–2.4% in wheat grain and bran [7]. NSP can increase digesta viscosity and reduce nutrient digestion [8]. Adding a multicarbohydrase (xylanase, β-glucanase, hemicellulase, and pectinase) to the diets can promote growth performance in nursery pigs [6,9]. Beyond the efficacy of individual enzymes, the supplementation of multicarbohydrase and phytase complex (MCPC) in diets fed to poultry species have been shown to allow important reductions in energy and nutrients without any adverse effects on the performance of the birds [10,11,12]. Meanwhile, as corn, wheat, and soybean are staple food grains for humans, their common application as primary conventional feedstuffs for animals directly competes with their allocation for human consumption, as well as increasing their cost [13]. Therefore, dietary supplementation of exogenous enzymes to improve the feed utilization efficiency could be a beneficial choice for the swine industry. However, to date, only limited information is available of the efficacy of multienzyme on performance, carcass traits, and meat quality in swine fed a diet with miscellaneous feedstuffs.

Since corn, soybean meal, wheat, and their byproducts are common feedstuffs, which contain abundance of phytate, arabinoxylan, and β-glucan [1,6,7], it was hypothesized that nutrient digestibility of diets based on these feedstuffs for pigs could be improved by supplemental enzyme products that target most of the NSP and phytate in the diet. The objective of the present study was to evaluate the efficacy of an MCPC that contains xylanase, β-glucanase, α-arabinofuranosidase, and phytase on growth performance, nutrient digestibility, carcass traits, and meat quality in growing and finishing pigs fed a corn-soybean meal-wheat and their byproducts-based diet deficient in net energy (NE), digestible amino acids (dig. AA), digestible P (dig. P), and calcium (Ca).

## 2. Materials and Methods

### 2.1. Animals, Experimental Design, and Diets

A total of 300 growing pigs [Duroc × Large White × Landrace; initial body weight (BW) = 25.3 ± 0.7 kg] were allotted in a randomized complete block design to 3 treatment groups, with 10 replicates of 10 pigs (5 barrows and 5 gilts) per pen. Pigs were fed positive control (PC), negative control (NC), or NC supplemented with MCPC (NC + MCPC) diets. The MCPC (Rovabio Advance Phy, Adisseo France S.A.S., France) supplied at least 1800, 1244, 6600, and 1000 units of xylanase, β-glucanase, α-arabinofuranosidase, and phytase per kilogram of diet, respectively. The units of these enzyme activities were defined according to Jlali et al. (2020) [14]. Specifically, one visco-unit of endo-1,4-β-xylanase activity is defined as the amount of enzyme reducing the viscosity of the solution, to give a change in relative fluidity of 1 dimensionless unit per minute per milliliter (or per gram) under the conditions of the assay (pH 5.5 and 30 °C). One azo-β-glucanase unit of endo-1,3(4)-β-glucanase activity is defined as the amount of enzyme releasing oligomers, which are soluble in ethanol, to give an absorbance of 0.820 units at 590 nm under the conditions of the assay (20 min at pH 4.6 and 30 °C). One unit of arabinofuranosidase refers to the amount of enzyme that releases 1 nmol of arabinose per min from the hydrolysis of wheat arabinoxylan in defined assay conditions (pH 4, 50 °C). Arabinose was quantified using high-performance anion-exchange chromatography with amperometric detection. One unit of phytase is defined as the amount of enzyme that liberates one micromole of inorganic orthophosphate from phytic acid per minute at pH 5.5 and 37 °C. The activities of xylanase and phytase in the diet were analyzed by ADISSEO France using the methods as described by described by European Food Safety Authority (EFSA) (2014) and ISO standard 30024 (2009) [15,16]. The doses were chosen on the basis of a previous study which reported that dietary supplementation of MCPC that contained these doses of enzymes can improve the nutrient digestibility in swine [17]. The PC diet was adequate in all nutrients according to National Research Council (NRC) (2012) recommendations, and the NC diet was the PC diet reduced in net energy (NE), standard ileal digestible amino acid (SID AA), standardized total tract digestible (STTD) P, and Ca by approximately 74 kcal/kg, 7%, and 0.134 percentage points, and 0.119 percentage points, respectively (Table 1). The doses were chosen based on the reports that the dietary supplementation of MCPC can compensate the performance of chicks fed the diet reduced in apparent metabolizable energy (74–85 kcal/kg), dig. AA (1.5–3.0%), digestible P (0.15–0.20 percentage points), and digestible Ca (0.12–0.16 percentage points), respectively [10,18]. The diets were fed in 4 growth phases based on BW: phase 1: 25–50 kg, phase 2: 50–75 kg, phase 3: 75–100 kg, and phase 4: 100–135 kg. The feed formulation software (Allix; A-Systems, Versailles, France) was used to estimate the nutrients values and formulate the diets. Pigs were allowed free access to the designated diets and water throughout the experiment. Body weight and feed intake were measured by phase for calculation of body weight gain (BWG), feed intake (FI), and feed/gain (F/G) ratio. Titanium dioxide (TiO_2_; 0.3%) was added as an indigestible marker to all diets of phase 4 during the last 2 weeks of feeding for nutrient digestibility determination.

### 2.2. Apparent Total Tract Digestibility Analysis

Fresh fecal samples from 10 pens (pooled from 10 pigs/pen) per treatment were collected daily for 2 days before slaughter (at the end of phase 4) and the corresponding feed for each pen was collected 1 day before fecal collection [19]. Fecal and feed samples were stored at −20 °C for subsequent measurement of apparent total tract digestibility (ATTD). Before chemical analysis, all feces from each treatment were thawed and mixed thoroughly before sampling, and they were dried to a constant weight in a 65 °C oven for analysis [8]. Diets and feces were ground to a particle size of less than 0.5 mm for analysis of gross energy, crude protein, crude fat, ash, P, Ca, and TiO_2_. All chemical measurements were conducted in duplicate according to Zhao et al. (2018) [20] and Sun et al. (2020) [6]. Briefly, the gross energy was determined by an adiabatic oxygen bomb calorimeter (Parr 6400, Parr Instrument Inc., Moline, IL, USA). The crude protein content was analyzed via measuring nitrogen according to the micro-Kjeldahl method [19]. The crude fat content was measured by diethyl ether extraction [21]. The ash content was determined by burning samples in a muffle furnace. The contents of Ca, P, and TiO_2_ were determined by a spectrophotometry method (UV-2401, Shimadzu Corp., Kyoto, Japan). The ATTD of the nutrients was calculated using the equation as described by Sun et al. (2020) [6].

### 2.3. Carcass Traits and Meat Quality

At the end of the feeding trial and after 12 h of feed deprivation, 10 male pigs (1 pig/pen) from each treatment were selected according to the treatment average BW for the analysis of the carcass traits and meat quality. The backfat thickness of the pigs was measured 65 mm from the left side of the dorsal midline at the last rib level (P2) by an ultrasound (Renco B-07, Minneapolis, MN, USA), according to Zhang et al. (2019) [22]. The selected pigs were weighed and sacrificed using electrical stunning (120 V, 200 Hz) and exsanguination. The carcass was weighted by the removal of the head, feet, tail, and viscera. The percentage of the carcass weight relative to live BW was calculated and expressed as the carcass rate. The loin eye area was traced over the exposed surface muscle area at the tenth rib and the area was calculated by a planimeter.

The *Longissimus dorsi* muscle between the sixth and seventh ribs was isolated from the right side of carcass for meat quality analysis [23,24]. Briefly, the muscle pH was measured in triplicate at 45 min and 24 h after slaughter using a pH meter (pH-Star, SFK-Technology, Copenhagen, Denmark). Meat color (L* = lightness, a* = redness, and b* = yellowness) was measured in triplicate at 45 min postmortem using a chroma meter (Minolta Camera, Osaka, Japan). Marbling score was evaluated using a 5-point hedonic scale: 1- very low in intramuscular fat and 5- very dark, very high in intramuscular fat. The fresh meat sample was cut into shaped strips (1 × 1 × 3 cm) and weighed and then placed in a Whirl-Pak bag (Nasco, Fort Atkinson, WI, USA). After this, the sample was reweighed after being hung in a 4 °C cooler for 24 h to calculate drip loss. The fresh meat sample was cut into shaped strips (1 × 1 × 3 cm) and weighed and sealed in a plastic bag, and then cooked in a water bath at 75 °C for 45 min. Then the sample was dried and reweighed to calculate cooking loss. Warner–Bratzler shear force was measured 6 times for each sample by a texture analyzer (Northeast Agricultural University, Harbin, China) with a 15 kg load transducer and a crosshead speed of 200 mm/min. Six replicates of each sample were measured.

### 2.4. Statistical Analysis

Statistical analysis was performed by SPSS, version 13. Data are presented as means ± SD. Dietary effects were determined by one-factor ANOVA with a significance level of *p* < 0.05, and the Tukey–Kramer test was used for multiple mean comparisons.

## 3. Results

### 3.1. Growth Performance

The analyzed activities of xylanase and phytase in NC + MCPC diets of phase 1–4 were close to the expected amounts supplemented (Table 2). Growth performance results are presented in Table 3. Compared with the PC, the BWG was reduced (*p* < 0.05) by 4.97%, 5.37%, 12.89%, and 6.04%, respectively, during phase 1, 2, 3, and 4 in the NC group. Compared with the PC, the NC reduced (*p* < 0.05) FI by 7.98% and 2.07%, respectively, during phase 3 and the entire experimental period. Compared with the PC, the NC also increased (*p* < 0.05) F/G ratio by 2.78%, 4.72%, 5.38%, and 4.36%, respectively, during phase 1, 2, 3, and the entire experimental period. Moreover, dietary supplementation of the MCPC (NC + MCPC) increased (*p* < 0.05) BWG by 15.20% and 4.03%, respectively, during 3 and 1–4 relative to the NC group. It also reduced (*p* < 0.05) F/G ratio by 3.38%, 6.31%, and 2.57%, respectively, during 2, 3, and 1–4 compared with the NC group. Although NC + MCPC group decreased (*p* < 0.05) FI by 4.29% during phase 2, it increased (*p* < 0.05) FI by 7.99% during phase 3, relative to the NC group. However, the BWG, FI, and F/G ratio of pigs did not differ (*p* > 0.05) between the 3 groups during phase 4.

### 3.2. Apparent Total Tract Digestibility (ATTD) of Nutrients

The dietary MCPC supplementation significantly affected the ATTD of crude protein, crude fat, ash, phosphorus, and calcium (Table 4). Specifically, the NC group decreased (*p* < 0.05) the ATTD of crude protein (7.02%), crude fat (16.89%), P (15.09%), and Ca (6.65%) relative to the PC group. Notably, compared with the NC group, dietary supplementation of the MCPC (NC + MCPC) increased (*p* < 0.05) the ATTD of crude protein (6.99%), crude fat (26.39%), ash (27.91%), P (34.92%), and Ca (7.83%).

### 3.3. Carcass Traits and Meat Quality

The effects of dietary treatment on carcass traits and meat quality are presented in Table 5. No significant difference was observed between the PC and the NC diets on carcass traits and meat quality parameters (*p* > 0.05). The addition of MCPC to the NC diet increased (*p* < 0.05) the loin eye area by 11.27%.

## 4. Discussion

In the current study, as expected, the NC diet deficient in energy and nutrients reduced the growth performance of growing and finishing pigs. Indeed, the NC diet deficient in NE, SID AA, digestible P, and Ca reduced BWG, FI, and(or) feed conversion during the growing/finishing phases 1, 2, 3, and 1–4. These results are in agreement with previous reports in which pigs fed diets deficient in nutrients, such as energy, AA, P, and(or) Ca, had lower performance outcomes [25,26,27,28]. Meanwhile, in the current study, the NC diet composed of corn, soybean meal, wheat and their byproducts contained antinutritional factors such as arabinoxylan, β-glucans, and phytate, which can reduce the nutrient digestibility and thus impair the growth performance of pigs [7,17,28,29]. The addition of MCPC, enriched in xylanase, β-glucanase α-arabinofuranosidase, and phytase, increased the BWG, FI, and(or) feed to gain ratio in phases 2, 3, and in the overall experimental period. This finding is similar with previous studies that showed that dietary supplementation of exogenous enzymes, NSP-degrading enzymes, and phytase in combination, improved the growth performance in poultry and pigs [10,11,25,27].

The NC diet was generated from the PC diet by a partial substitution of corn and soybean meal in the PC diet with wheat bran and soy hulls in the present study. The wheat bran and soy hulls have a greater content of NSP than corn and soybean meal [30,31,32], which might help to increase the opportunity to show the enzyme effects in the current study. Since pigs as monogastric animals that do not digest well, NSP and the negative effects of dietary NSP content on nutrients and energy digestibility had been demonstrated previously [33,34,35,36], where the ATTD of crude protein, crude fat, gross energy, ash, P, and Ca for the NC diet was lower than that for the PC diet. However, the ATTD of gross energy and ash values were only numerically lower with the NC diet than those with the PC diet.

Supplementation of the NC diet with MCPC improved the ATTD of crude protein, crude fat, ash, P, and Ca of pigs. As described above, the NC diet contains antinutritional factors: (1) the NSPs can increase digesta viscosity [8,28] and (2) phytate can chelate with minerals and protein forming insoluble complexes [5,37], which reduce the nutrient digestibility in monogastric animals. In the present study, adding exogenous enzymes to the NC diet, including NSP-degrading enzymes and phytase, can hydrolyze NSP and phytate and thus liberate more nutrients to the digestive tract and increase the ATTD of nutrients in pigs [27,37]. These outcomes are in agreement with previous studies that reported dietary supplemental exogenous enzymes multicarbohydrase and(or) phytase increased digestibility of nutrients and performance of pigs fed the corn, wheat, soybean, and(or) their byproducts-based diets [6,9,25,27,28,38,39]. Nevertheless, commercial feeds usually contain less wheat bran and soy hulls than those of the NC diet in the present study, which might narrow the effects of the MCPC in practical applications.

The NC diet did not affect the carcass and meat quality traits. These results were surprising as the NC diet has higher fiber or NSP and lower NE and crude fat, which can decrease fat digestibility, and consequently may also reduce the fat synthesis, decreasing the backfat thickness and marbling score and other carcass traits in pigs [40,41,42]. Kerr et al. (1995) [43] reported that diets reduced in crude protein levels with inadequate amino acids can increase the body fat content in pigs. However, the NC diet used in this study was formulated with a high level of fiber and low content of crude fat, compared with an adequate-nutrient diet, which probably limited the increase of body fat content in animals. The addition of MCPC to the NC diet only increased the loin eye area relative to those of the NC group. Since the loin eye area is positively correlated with the carcass weight of pigs [44], the increase of the loin eye area observed with the NC + MCPC diet was certainly due to increase of the slaughter weight of the pigs relative to those fed the NC diet. Meanwhile, the NC dietary MCPC supplementation did not affect any meat quality parameters, which was consistent with previous reports in pigs [41,45]. Reducing NE, dig. AA, dig. P, and Ca and utilization of co-products and MCPC in growing-finishing diets results in reducing the production cost. Using the data obtained from this study, the production cost was reduced by approximately 7% compared to that with the PC diet, indicating therefore an important financial advantage by using MCPC in growing-finishing pigs.

## 5. Conclusions

This study showed that MCPC supplementation, enriched in xylanase, α-arabinofuranosidase, β-glucanase, and phytase, improved nutrients digestibility, thus allowing pigs to reach a growth performance similar to animals fed a diet adequate in all nutrients in a more economical way. Thus, NE, digestible AA, and P can be lowered by 74 kcal/kg, 7.0% and 0.134% unit, respectively, in MCPC-supplemented diets without effects on feed efficiency and reducing the production cost of pigs during the growing-finishing period.

## Figures and Tables

**Table 1:** Composition and nutritional values of the diets.

Ingredient %	Phase 1: 20–50 kg		Phase 2: 50–75 kg		Phase 3: 75–100 kg		Phase 4: 100–135 kg	
	PC	NC	PC	NC	PC	NC	PC	NC
Corn	59.05	55.86	63.76	60.16	67.88	64.68	69.02	65.06
Soybean meal	13.95	11.28	9.95	7.60	6.42	3.62	5.50	3.30
Wheat bran	10.00	15.00	10.00	15.20	10.00	15.40	10.00	15.60
Wheat	10.00	10.00	10.00	10.00	10.00	10.00	10.00	10.00
Soy hulls	1.00	3.00	1.26	3.10	1.50	3.20	1.87	3.40
Soybean oil	2.57	2.18	2.00	1.65	1.50	1.09	1.38	1.06
CaCO3	0.82	0.78	0.73	0.68	0.62	0.57	0.53	0.44
MCP ^1^	0.93	0.31	0.78	0.16	0.67	0.05	0.54	-
L-Lys HCl 98	0.57	0.54	0.52	0.49	0.46	0.46	0.33	0.32
DL-Met 99	0.11	0.09	0.07	0.05	0.04	0.02	-	-
L-Thr	0.16	0.15	0.14	0.13	0.12	0.12	0.07	0.06
L-Trp	0.06	0.05	0.05	0.05	0.05	0.05	0.03	0.03
L-Val	0.05	0.04	0.02	0.01	-	-	-	-
Premix ^2^	0.50	0.50	0.50	0.50	0.50	0.50	0.50	0.50
NaCl	0.23	0.23	0.23	0.24	0.24	0.24	0.24	0.24
Nutrient values ^3^, %								
Crude protein	14.56	14.01	13.04	12.62	11.71	11.17	11.23	10.91
Crude fat	5.50	5.17	5.05	4.74	4.64	4.28	4.55	4.27
Crude fiber	3.62	4.53	3.57	4.46	3.53	4.37	3.63	4.45
Ash	4.33	3.93	3.94	3.55	3.58	3.18	3.36	3.01
NE, kcal/kg	2475	2401	2475	2401	2475	2401	2475	2401
SID Lys	0.98	0.91	0.85	0.79	0.73	0.68	0.61	0.57
SID Met	0.32	0.29	0.27	0.24	0.22	0.20	0.18	0.17
SID Met + Cys	0.55	0.51	0.48	0.45	0.42	0.39	0.38	0.37
SID Thr	0.59	0.55	0.52	0.48	0.46	0.43	0.40	0.37
SID Trp	0.17	0.16	0.15	0.14	0.13	0.12	0.11	0.10
SID Arg	0.78	0.73	0.67	0.63	0.58	0.53	0.55	0.52
SID His	0.34	0.32	0.31	0.29	0.28	0.26	0.27	0.26
SID Ile	0.51	0.47	0.45	0.42	0.40	0.36	0.38	0.36
SID Leu	1.17	1.10	1.10	1.04	1.04	0.97	1.02	0.96
SID Val	0.64	0.59	0.55	0.51	0.48	0.45	0.47	0.44
Ca	0.66	0.54	0.59	0.47	0.52	0.40	0.46	0.34
Total P	0.58	0.47	0.53	0.43	0.50	0.39	0.47	0.38
STTD P	0.31	0.18	0.27	0.14	0.24	0.11	0.21	0.09
Sodium	0.10	0.10	0.10	0.10	0.10	0.10	0.10	0.10

^1^ MCP, monocalcium phosphate; NE, net energy; SID, Standard ileal digestible; STTD, standardized total tract digestible; Ca, calcium; P, phosphorus. ^2^ Premix, vitamin and mineral provided /kg diet: retinyl acetate, 10,000 IU; cholecalciferol 2500 IU; dl-α-tocopherol acetate, 50 IU; menadione, 5.0 mg; thiamin, 2.0 mg; riboflavin, 5.0 mg; pantothenic acid, 12.0 mg; pyridoxine, 10.0 mg; niacin, 30.0 mg; biotin, 0.2 mg; folic acid, 1.5 mg; cyanocobalamin 0.05 mg; choline chloride 1500 mg; iron, 100 mg; copper, 20 mg; manganese, 25 mg; zinc, 100 mg; selenium, 0.3 mg; iodine, 0.3 mg. ^3^ The analyzed dry matter, crude protein and gross energy content in the positive control (PC) and negative control (NC) diets of the Phase 4 are 86.1% and 86.5%, 11.85% and 11.10%, and 4221 and 4312 Kcal/kg, respectively. All the others are calculated values.

**Table 2:** The analyzed activities of xylanase and phytase in NC + MCPC diet.

Period	Xylanase, U	Phytase, U
Phase 1	1890	1140
Phase 2	2106	1300
Phase 3	2106	1400
Phase 4	1674	1040

**Table 3:** Effect of multicarbohydrase and phytase complex on growth performance of growing-finishing pigs ^1^.

Item	PC	NC	NC + MCPC	*p*-Value
Initial body weight, kg	25.28 ± 0.42	25.19 ± 0.37	25.34 ± 0.33	0.67
Phase 1: 25–50 kg	-	-	-	-
BWG, kg	24.36 ± 0.72 ^a^	23.15 ± 1.22 ^b^	23.06 ± 1.37 ^b^	0.02
FI, kg	52.63 ± 2.70	51.42 ± 2.02	52.24 ± 2.61	0.42
F/G, kg/kg	2.16 ± 0.07 ^b^	2.22 ± 0.05 ^a^	2.27 ± 0.09 ^a^	0.01
Phase 2: 50–75 kg	-	-	-	-
BWG, kg	27.21 ± 1.27 ^a^	25.75 ± 1.43 ^b^	25.46 ± 1.73 ^b^	0.03
FI, kg	69.07 ± 2.99 ^a^	68.33 ± 2.40 ^a^	65.40 ± 2.92 ^b^	0.01
F/G, kg/kg	2.54 ± 0.12 ^b^	2.66 ± 0.12 ^a,^*	2.57 ± 0.09 ^b,^*	0.04
Phase 3: 75–100 kg	-	-	-	-
BWG, kg	23.19 ± 2.33 ^a^	20.20 ± 1.94 ^b^	23.27 ± 1.38 ^a^	<0.01
FI, kg	72.92 ± 4.70 ^a^	67.10 ± 5.10 ^b^	72.46 ± 3.49 ^a^	<0.01
F/G, kg/kg	3.16 ± 0.22 ^b,#^	3.33 ± 0.22 ^a,#^	3.12 ± 0.21 ^b^	0.09
Phase 4: 100–135 kg	-	-	-	-
BWG, kg	32.31 ± 2.39	31.50 ± 2.65	32.87 ± 2.97	0.91
FI, kg	124.79 ± 5.73	125.95 ± 6.07	126.65 ± 8.35	0.82
F/G, kg/kg	3.87 ± 0.25	4.01 ± 0.24	3.87 ± 0.26	0.97
Phase 1–4: 25–135 kg	-	-	-	-
BWG, kg	107.07 ± 3.01 ^a,+^	100. 60 ± 2.64 ^c^	104.65 ± 2.79 ^b,+^	<0.01
FI, kg	319.41 ± 9.47 ^a,-^	312.81 ± 11.35 ^b,-^	316.76 ± 12.07 ^a,b^	0.23
F/G, kg/kg	2.98 ± 0.09 ^b^	3.11 ± 0.08 ^a^	3.03 ± 0.6 ^b^	0.01

^1^ Results are reported as the mean ± SD, *n* = 10. Labeled means in a row without a common superscript letter differ, *p* < 0.05. A given 2 means within the same plot labeled with *, #, + or - differ at *p* = 0.06–0.1. BWG, body weight gain; FI, feed intake; F/G, feed to gain ratio; PC, positive control; NC, Negative control; NC + MCPC, negative control diet supplemental with a multicarbohydrase and phytase complex supplying at least 1800, 1244, 6600, and 1000 units of xylanase, β-glucanase, α-arabinofuranosidase, and phytase per kilogram of diet, respectively.

**Table 4:** Effect of multicarbohydrase and phytase complex on the apparent total tract digestibility of gross energy (GE), crude protein, crude fat, ash, P, and Ca of pigs ^1^.

Item	PC	NC	NC + MCPC	*p*-Value
Gross energy, %	82.1 ± 2.3	80.0 ± 3.6	81.8 ± 1.7	0.16
Crude protein, %	76.9 ± 2.8 ^a^	71.5 ± 4.9 ^b^	76.5 ± 2.9 ^a^	<0.01
Crude fat, %	73.4 ± 5.6 ^a^	61.0 ± 8.8 ^b^	77.1 ± 6.0 ^a^	<0.01
Ash, %	33.0 ± 6.4 ^b^	32.6 ± 7.9 ^b^	41.7 ± 6.6 ^a^	0.01
P, %	57.0 ± 11.4 ^b^	48.4 ± 9.2 ^c^	65.3 ± 6.2 ^a^	<0.01
Ca, %	75.2 ± 3.2 ^a^	70.2 ± 5.4 ^b^	75.7 ± 3.3 ^a^	0.01

^1^ Results are reported as the mean ± SD, *n* = 10. Labeled means in a row without a common superscript letter differ, *p* < 0.05. Ca, calcium; P, phosphorus; PC, positive control; NC, Negative control; NC + MCPC, negative control diet supplemental with a multicarbohydrase and phytase complex supplying at least 1800, 1244, 6600, and 1000 units of xylanase, β-glucanase, α-arabinofuranosidase, and phytase per kilogram of diet, respectively.

**Table 5:** Effect of the multicarbohydrase and phytase complex on the carcass traits and meat quality of pigs ^1^.

Item	PC	NC	NC + MCPC	*p*-Value
Carcass traits	-	-	-	-
Backfat thickness, mm	27.1 ± 3.3	26.1 ± 2.3	26.9 ± 2.3	0.68
Carcass yield, %	72.7 ± 1.9	72.2 ± 1.9	73.4 ± 1.6	0.38
Loin eye area, cm^2^	43.9 ± 3.2 ^a,b^	40.8 ± 2.0 ^b^	45.4 ± 5.1 ^a^	0.04
Meat quality	-	-	-	-
Marbling score	2.5 ± 1.0	2.1 ± 0.7	2.3 ± 0.5	0.52
Meat color	-	-	-	-
L*	57.9 ± 9.7	60.5 ± 6.4	58.6 ± 5.9	0.73
a*	17.9 ± 1.2	18.4 ± 0.6	17.9 ± 1.7	0.61
b*	6.75 ± 1.12	6.35 ± 0.70	6.22 ± 1.41	0.55
pH_45min_	6.25 ± 0.05	6.32 ± 0.12	6.32 ± 0.07	0.17
pH_24h_	5.55 ± 0.34	5.52 ± 0.19	5.61 ± 0.24	0.79
Drip loss, %	6.33 ± 2.15	5.40 ± 0.99	5.00 ± 2.71	0.36
Cooking loss, %	37.6 ± 5.9	38.7 ± 3.1	39.4 ± 2.4	0.63
Shear force, N	16.1 ± 4.9	17.0 ± 5.5	15.4 ± 4.0	0.76

^1^ Results are reported as the mean ± SD, *n* = 10. Labeled means in a row without a common superscript letter differ, *p* < 0.05. PC, positive control; NC, Negative control; NC + MCPC, negative control diet supplemental with a multicarbohydrase and phytase complex supplying at least 1800, 1244, 6600, and 1000 units of xylanase, β-glucanase, α-arabinofuranosidase, and phytase per kilogram of diet, respectively, L* = lightness, a* = redness, and b* = yellowness.

## Data Availability

Data available in a publicly accessible repository.
